# BMS-708163 and Nilotinib restore synaptic dysfunction in human embryonic stem cell-derived Alzheimer’s disease models

**DOI:** 10.1038/srep33427

**Published:** 2016-09-19

**Authors:** Hisae Nishioka, Norie Tooi, Takehisa Isobe, Norio Nakatsuji, Kazuhiro Aiba

**Affiliations:** 1 Institute for Integrated Cell-Material Sciences (WPI-iCeMS), Kyoto University, Kyoto 606-8501, Japan; 2 Graduate School of Medicine, Kyoto University, Kyoto 606-8501, Japan; 3 Institute for Frontier Medical Sciences, Kyoto University, Kyoto 606-8507, Japan

## Abstract

Alzheimer’s disease (AD) is the most common form of dementia. Cellular AD models derived from human pluripotent stem cells are promising tools in AD research. We recently developed human embryonic stem cell-derived AD models which overexpress mutant Presenilin1 genes, and which exhibit AD phenotypes, including synaptic dysfunction. In this study, we found that our AD models showed reduced levels of RAB3A and SV2B proteins in the pre-synapses, which is a possible cause of electrophysiological abnormalities. Through the screening of chemical compounds using our AD models, we have identified Aβ peptide inhibitors which decrease the concentration of Aβ in culture supernatant. Among these, BMS-708163 and Nilotinib were found to improve the expression levels of RAB3A and SV2B proteins and to recover the electrophysiological function in our AD models. These results suggest that the AD models we developed are promising materials for the discovery of AD drugs that target the expression of pre-synaptic proteins and synaptic function.

Alzheimer’s disease (AD) is the most common form of dementia in the elderly population. Cerebral atrophy and senile plaques can be found in the AD patient’s brain, where neural dysfunction and neural death are observed^1^,^2^. An increase in the amyloid-β 42 (Aβ42) and Aβ43 ratio relative to Aβ40 is thought to be involved in plaque generation^3^,^4^.

AD can be categorized into sporadic and familial types: familial AD (FAD) is mainly associated with mutations of three genes (the amyloid precursor protein [APP] gene, the presenilin 1 [PS1] gene and the presenilin 2 [PS2] gene)^5^,^6^. The PS1 and PS2 proteins are related to the enzyme activity of γ-secretase that produces Aβ from APP^7^,^8^,^9^. In the process of Aβ generation, mutations in APP, PS1 or PS2 are involved in the increase of Aβ42 and Aβ43. These increases are thought to promote Aβ oligomerization and lead to dementia^10^,^11^,^12^. Thus, the inhibition of Aβ secretion is believed to represent a potential avenue for AD treatment. However, at the present time Aβ inhibitors have not progressed through clinical trials.

Recently, AD patient-specific induced pluripotent stem cell (iPSC) lines (AD-iPSCs) have been established as cellular models of AD (reviewed in ref. 13). AD-iPSCs have potential application in the discovery of new drugs and the investigation of disease mechanisms^14^,^15^. AD-iPSC-derived neurons show increased amounts of Aβ42 and increased Aβ42/Aβ40 ratios as AD phenotypes, but do not reproduce the electrophysiological abnormalities of AD^16^,^17^,^18^,^19^,^20^,^21^,^22^. In addition, it is difficult to accurately compare disease iPSCs and healthy iPSCs because they have different genomic backgrounds. Although healthy isogenic iPSCs can be generated from disease iPSCs with the use of genome-editing technologies^23^,^24^, it is not a cost-effective method for generating multiple isogenic iPSC lines from a single cell line.

We recently established human embryonic stem cell (hESC) lines which overexpress a mutant PS1 with the use of a site-directed integration system^25^. This gene integration system enables to generate cell lines with identical genetic backgrounds. We found that neurons derived from these hESCs showed AD phenotypes, including increased Aβ42/Aβ40 ratios and spontaneous excitatory postsynaptic current (sEPSC) abnormalities^26^.

In the present study, we identified a cause of the electrophysiological abnormality in our AD models which overexpressed mutant PS1, screened the FDA-approved chemical libraries using our AD models, and examined whether the application of Aβ inhibitors can lead to the recovery of synaptic dysfunction.

## Results

### The decrease of RAB3A and SV2B proteins in the cellular models of AD

**Figure 1:**
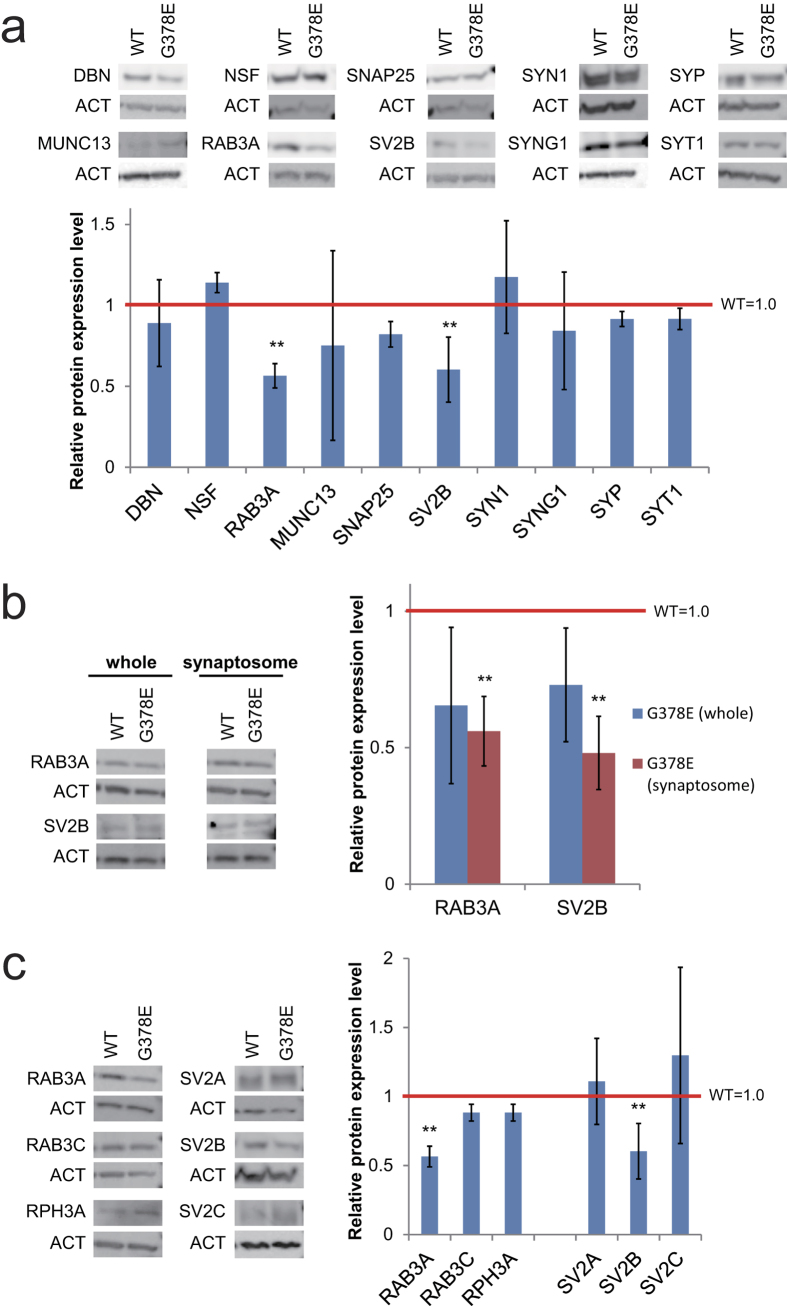
The decrease of RAB3A and SV2B protein levels in the pre-synapses of PS1-G378E neurons. Immunoblot analyses of the pre-synaptic proteins in the synaptosomes of PS1-G378E neurons (**a**) RAB3A and SV2B proteins in the whole fraction or synaptosomes of PS1-G378E neurons (**b**) and the RAB3 and SV2 family proteins in synaptosomes (**c**). β-actin (ACT) was used as an internal control. Each protein level in the PS1-WT neurons was defined as 1.0. **P < 0.01, as determined by Mann–Whitney U test. Neural differentiation and subsequent synaptosome preparation were independently performed four times (n = 4). Supplementary Fig. S4 shows that isolation of synaptosomes was successfully carried out in all preparations. Mean ± SD. WT, PS1-wild type neurons; G378E, PS1-G378E neurons; DBN, drebrin; NSF, N-ethylmaleimide sensitive factor; RPH3A, rabphilin 3A; SNAP25, synaptosome associated protein 25 kDa; SV2, synaptic vesicle glycoprotein 2; SYN1, synapsin 1; SYNG1, synaptogyrin 1; SYT1, synaptotagmin 1; SYP, synaptophysin.

As described in our previous paper, our AD model (neurons overexpressing PS1-G378E) showed the reduction of sEPSC; however, the action potential amplitude and frequency did not differ between the neurons overexpressing PS1-G378E (PS1-G378E neurons) and those overexpressing wild-type PS1 (PS1-WT neurons)^26^. These facts suggested that a normal function of the post-synapses and an abnormal function of the pre-synapses in the PS1-G378E neurons. To elucidate the cause of electrophysiological abnormality in the pre-synapses of our AD models, we examined whether there is a difference in the level of pre-synaptic proteins in PS1-G378E and PS1-WT neurons. Western blot analyses were carried out using an enriched fraction of synaptic proteins (synaptosomes). We examined 10 pre-synaptic proteins, and found an approximately 40% decrease in the expression levels of RAB3A and SV2B proteins in synaptosomes from PS1-G378E neurons in comparison to PS1-WT neurons (Fig. 1a), while their gene expression levels in PS1-G378E and PS1-WT neurons did not differ to a statistically significant extent; there was no difference in the expression of non-affected proteins such as SYP (synaptophysin) and SYT1 (synaptotagmin 1) (Supplementary Fig. S1). Furthermore, using whole cell extracts, we found that, although a decreasing tendency was found, the RAB3A and SV2B levels did not differ to a statistically significant extent in PS1-G378E and PS1-WT neurons (Fig. 1b). These results suggest that our AD model might normally regulate the gene expression of RAB3A and SV2B, but be defective in the translation and/or localization of the pre-synapses of these proteins. Next, we examined whether there were any differences in the levels of other RAB3 or SV2 family proteins (Fig. 1c). The results of a Western blot analysis showed that the levels of RAB3C, RPH3A (rabphilin 3A), SV2A and SV2C from PS1-G378E neurons did not differ from those of PS1-WT neurons, indicating that RAB3A and SV2B are the only proteins from the RAB3 and SV2 families, respectively, that are abnormally expressed in the pre-synapses of our model.

### The screening of Aβ40 inhibitors using AD model neurons

**Figure 2:**
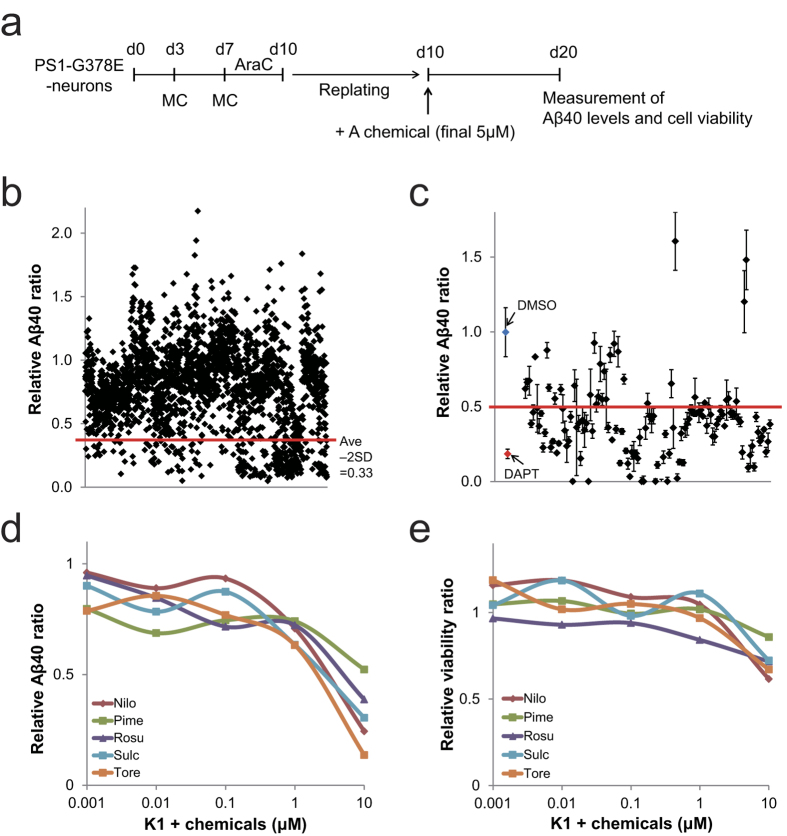
The screening of Aβ40 inhibitors using AD models. (**a**) Scheme showing the cell culture procedure of chemical screening. (**b**) The result of first screening of Aβ40 inhibitors. The red line shows the value of the average ratio minus double the standard deviation (Ave - 2SD = 0.33). The compounds that reduced the Aβ40 ratio below Ave - 2SD value (0.33) passed the first screening. The Aβ40 level of DMSO treatment was considered to be 1.0. (**c**) The result of second screening in Aβ40 inhibitors. The red line indicates the criterion of hit compounds in the second screening (=0.5). (**d,e**) The effects of Aβ inhibition (**d**) and cell survival (**e**) in dose-response experiments using K1-derived neurons. These results show that PS1-overexpression did not influence the screening of Aβ inhibitors. The numerical data is shown in Supplementary Table S2. The amount of Aβ40 and cell viability in DMSO-treated PS1-G378E or K1 neurons was defined as 1.0. *P < 0.05, as determined by Steel’s test. Three independent experiments, each time in triplicates were performed (n = 3). Values are Mean ± SD. AraC, cytosine arabinoside; G378E, PS1-G378E neurons; K1, KhES-1-derived neurons; Nilo, Nilotinib; Pime, Pimecrolimus; Rosu, Rosuvastatin Calcium; Sulc, Sulconazole Nitrate salt; Tore, Toremifene Base.

Our AD models showed increased Aβ42 and Aβ43 levels^26^. The increase of Aβ42 and Aβ43 could have resulted in the reduction of RAB3A and SV2B protein levels in the pre-synapses of our AD model. Thus, the inhibition of Aβ production and secretion may have the potential to restore the RAB3A and SV2B protein levels in the pre-synapses, and recover synaptic dysfunction in PS1-G378E neurons. We developed a screening system for our AD models (Fig. 2a) and screened chemicals that block Aβ secretion and production using libraries of known chemicals, including FDA-approved chemicals (see Methods). In the first screening, 246 out of 2408 chemicals tested were effective at reducing Aβ secretion passed (Fig. 2b). All of the γ-secretase inhibitors in the libraries were effective, including N-[N-(3,5-difluorophenacetyl-L-alanyl)]-S-phenyglycine t-butyl ester (DAPT), LY-450139 (Semagacestat) and BMS-708163 (Avagacestat)^27^,^28^,^29^ (Supplementary Table S1), indicating that our AD model may be used in the discovery of novel γ-secretase inhibitors. In the second screening, only 127 chemicals were used out of 246 chemicals because overlapping chemicals in both libraries, known Aβ inhibitors including β- and γ-secretase inhibitors, and toxic chemicals were removed. After 2^nd^ screening, 95 hit chemicals were identified (Fig. 2c).

Of 95 hit compounds, several FDA-approved drugs, including chemicals that have been previously reported as Aβ inhibitors^30^,^31^ were identified (Supplementary Table S1). Five of these FDA-approved drugs include: Nilotinib (a Bcr-Abl inhibitor), Pimecrolimus (a calcineurin inhibitor), Rosuvastatin Calcium (a HMG-CoA reductase inhibitor), Sulconazole Nitrate salt (an imidazole derivative) and Toremifene Base (a selective estrogen receptor modulator) were chosen for further experiments. These chemicals decreased the level of Aβ40, but did not affect viability of neurons.

Next, to confirm that PS1-overexpression did not influence the screening of Aβ inhibitors, we examined whether the five FDA-approved drugs inhibited Aβ generation against neurons differentiated from KhES-1 (K1), a normal hESC line. All of these drugs were found to inhibit Aβ in a dose-dependent manner without toxicity to the K1-derived neurons at concentrations of less than 10 μM (Fig. 2d,e and Supplementary Table S2). Therefore, PS1-overexpression did not affect the screening, indicating that our AD model, which overexpresses PS1, might be a promising cell source material for AD drug screening.

### BMS-708163 and Nilotinib restored RAB3A and SV2B proteins in the pre-synapses

**Figure 3:**
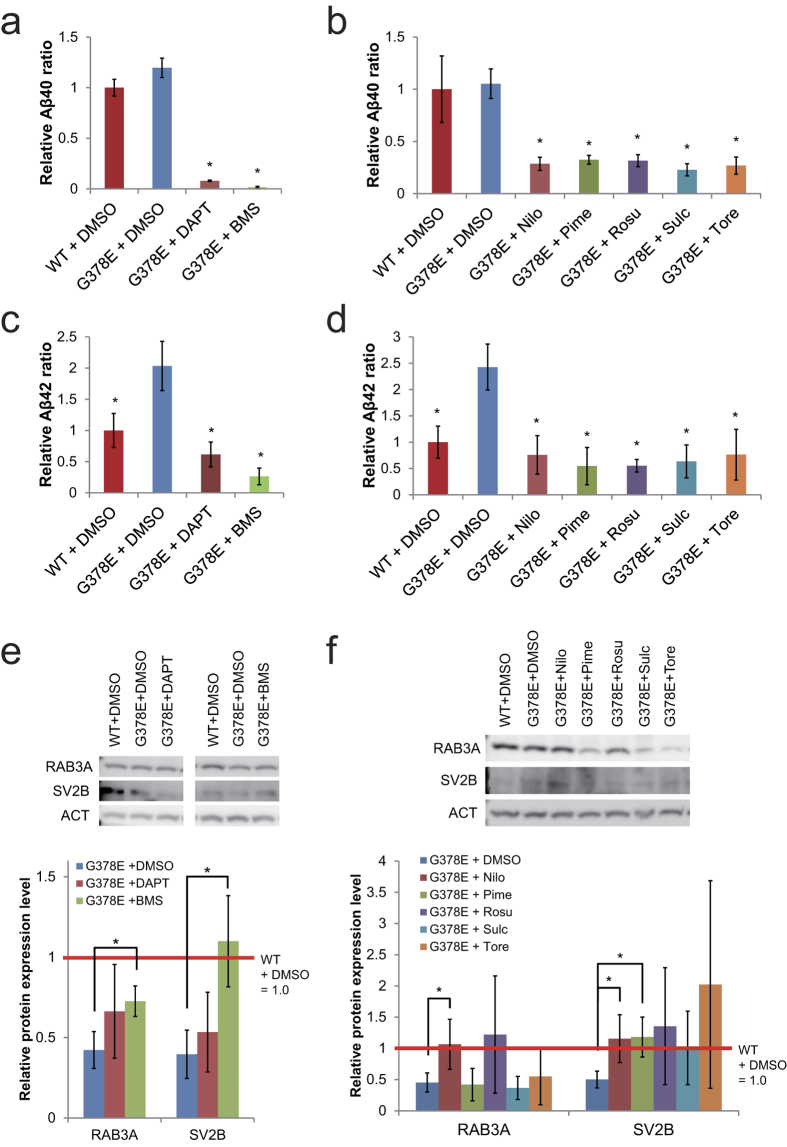
The recovery of RAB3A and SV2B protein levels in the pre-synapses of PS1-G378E neurons by γ-secretase inhibitors and five-hit compounds. (**a**,**b**) Aβ40 inhibition by γ-secretase inhibitors, DAPT and BMS-708163 (BMS) (**a**) and 5-hit compounds: Nilotinib (Nilo), Pimecrolimus (Pime), Rosuvastatin Calcium (Rosu), Sulconazole Nitrate salt (Sulc) and Toremifene Base (Tore) (**b**). (**c**,**d**) Aβ42 inhibition by γ-secretase inhibitors (**c**) and 5-hit compounds (**d**). The amount of Aβ40 and Aβ42 in PS1-WT neurons treated with DMSO was defined as 1.0. *P < 0.05, as determined by Steel’s test in comparison to PS1-G378E neurons treated with DMSO. Four independent experiments, each time in triplicates were performed (n = 4). Mean ± SD. (**e**,**f**) An immunoblot analysis of RAB3A and SV2B proteins in the synaptosomes of PS1-G378E neurons treated with γ-secretase inhibitors (**e**) or 5-hit compounds (**f**). β-actin (ACT) was used as an internal control. Each protein level in the DMSO-treated PS1-WT neurons was defined as 1.0. *P < 0.05, as determined by Steel’s test in comparison to DMSO-treated PS1-G378E neurons. The experiment was independently performed four times (n = 4). Mean ± SD. WT, PS1-wild type neurons; G378E, PS1-G378E neurons.

We next addressed whether the decrease of Aβ peptides led to the restoration of the expression levels of the RAB3A and SV2B proteins in the pre-synapses of our AD model. PS1-G378E neurons were treated with DAPT, BMS-708163, and 5 FDA-approved drugs in a different culture conditions from that used for screening. Even in different culture condition (see Methods), γ-secretase inhibitors and the 5 FDA-approved drugs decreased the Aβ40 level (Fig. 3a,b) and the Aβ42 level in our AD model (Fig. 3c,d). The levels of RAB3A and SV2B proteins were evaluated using synapotosomes from PS1-G378E neurons that were treated with these chemicals (Fig. 3e,f). The levels of RAB3A and SV2B proteins were increased 2 and 3-fold, respectively, by treatment with BMS-708163 (although RAB3A did not recover fully), but not by treatment with DAPT (Fig. 3e). Among the FDA-approved drugs, Nilotinib fully recovered the expression levels of the RAB3A and SV2B proteins (Fig. 3f). Pimecrolimus could restore the SV2B expression level, but not the RAB3A level. Others did not increase the protein expression levels to a statistically significant extent. These data suggest that the reduction of Aβ peptides alone was not sufficient to recover the RAB3A and SV2B proteins.

### BMS-708163 and Nilotinib recovered the synaptic dysfunction in PS1-G378E neurons

**Figure 4:**
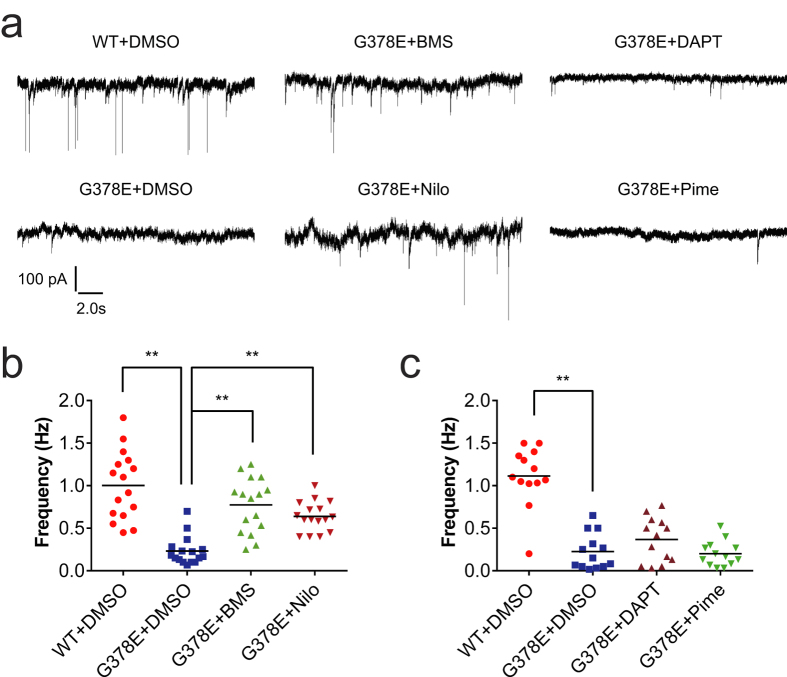
The recovery of synaptic dysfunctions in PS1-G378E neurons by BMS-708163 and Nilotinib. (**a**) Representative sEPSC recordings from PS1-WT and PS1-G378E neurons cultured with DMSO and each of the chemicals, respectively. (**b**,**c**) The frequencies of sEPSCs after the chemical treatments that led to the recovery of the level of the RAB3A and SV2B proteins (**b**) and those that did not (**c**). **P < 0.01, as determined by Steel’s test in comparison to DMSO-treated PS1-G378E neurons. The black bars represent the median values. A dot indicates the sEPSC frequency of a single neuron. The sample sizes of (**b**) and (**c**) were n = 16 and 13 neurons, respectively. Four independent neural differentiation experiments were carried out, and sEPSC were measured using 3 or 4 neurons from each differentiation. See the legends of Figs 2 and 3 for abbreviations.

The restoration of RAB3A and SV2B proteins led us to examine whether BMS-708163 and Nilotinib could recover the synaptic dysfunction in PS1-G378E neurons. As reported in our previous paper^26^, patch-clamp analyses showed that the sEPSC frequency in PS1-G378E neurons was lower than that in PS1-WT neurons (Fig. 4a). There was an approximately 3-fold increase in the sEPSC frequencies in PS1-G378E neurons that were treated with BMS-708163 or Nilotinib in comparison to DMSO-treated neurons (Fig. 4b). We also investigated the effects of DAPT and Pimecrolimus, which did not restore the RAB3A and SV2B protein levels (Fig. 4a,c). These treatments did not increase the sEPSC frequencies in PS1-G378E neurons. These data suggest that the restoration of both RAB3A and SV2B protein levels by BMS-708163 or Nilotinib might be required for recovering the sEPSC frequencies in our AD model.

## Discussion

It is known that the levels of pre-synaptic proteins are downregulated in the brains of AD patients with cognitive impairment in comparison to the brains of healthy individuals^32^. The level of RAB3A protein in the hippocampus and frontal cortex decreases in the brains of AD patients, while the total levels of SV2 proteins (including SV2A, 2B and 2C) in the brains of AD patients do not differ to a statistically significant extent from those in the brains of healthy individuals^32^. Consistently, the level of RAB3A protein was decreased in the synapse-enriched fraction of our AD model; however it was not reduced in the whole protein fraction. We found that our AD model showed a decrease of the levels of SV2B but not of SV2A or SV2C proteins. If our AD model mimicked the molecular features of AD phonotypes, the amount of SV2B protein might be reduced in the brains of AD patients. A study using an SV2B-specific antibody would be able to clarify this question.

Previous studies have shown decreases in the levels of other pre-synaptic proteins, such as SYP and SYT, in the brains of AD patients^32^. However, their expression levels were not decreased in our AD models. It is likely that this difference might be due to the different cellular conditions. Neurons are already lost in the AD brains, while our AD models did not show cell death. Hence, our AD model might mimic an early stage of AD progression (synaptic injury/dysfunction is supposed to be an early event in AD)^33^.

In the present study, we demonstrated that our AD model was useful in the chemical screening of previously reported Aβ inhibitors, including γ-secretase inhibitors and HMG-CoA reductase inhibitors^27^,^28^,^29^,^30^,^31^,^34^,^35^,^36^. Among these chemicals, except γ-secretase inhibitors, the molecular mechanisms of action as Aβ inhibitors are still unclear, but an upregulation of Aβ degrading enzymes such as neprilysin might be involved^37^. Our AD models showed increased Aβ42 and/or Aβ43 ratios, and synaptic abnormalities^26^. Hence, our AD models might be useful in applications to discover of Aβ42/43 modulators and drugs that can recover synaptic dysfunction.

In addition, we showed that both BMS-708163 and Nilotinib were able to restore synaptic dysfunction in our cellular AD models. BMS-708163 is a γ-secretase inhibitor^28^, and Nilotinib is an FDA-approved drug that is used for the treatment of adult leukemia^38^,^39^. A recent paper reported that tyrosine kinase inhibitors (Nilotinib and Bosutinib) enhanced amyloid clearance and cognitive performance in a mouse model of AD^40^. In addition, BMS-708163 and Nilotinib can cross the blood-brain barrier^28^,^41^. They are therefore expected to be efficacious in the treatment of AD. However, BMS-708163 has not passed clinical trials^42^. To date, all of the Aβ inhibitors have failed in clinical trials involving AD patients. It is thought that the treatments using Aβ inhibitors were administered too late to prevent neuronal loss in the brains of AD patients with dementia^43^. Thus, the administration of Aβ inhibitors to patients with preclinical AD or mild cognitive impairment (MCI) may cure their AD^44^. Our results suggest that BMS-708163 and Nilotinib might be effective for preventing synaptic dysfunction in preclinical AD or MCI patients.

Our study used neurons derived from PS1-G378E overexpressing cells along with PS1-WT hESCs. AD is believed to affect cholinergic neurons^45^. However, we did not use cholinergic neurons that were highly efficiently differentiated from PS1-overexpressing hESCs in this study. Only a few cell populations (0.9 ± 0.7%) were choline acetyltransferase-positive neurons in our hESC-derived neurons (Supplementary Fig. S2). Future research should confirm these drug effects using cholinergic neurons that were induced by direct differentiation methods (for example^46^,^47^).

The present study could not reveal the mechanism by which the application of BMS-708163 and Nilotinib led to an increase in the pre-synaptic protein levels and a recovery from electrophysiological abnormalities. Aβ inhibition alone cannot achieve these effects. Hence, these drugs might affect an unknown target that influences protein expression. Future studies should address the mechanism underlying the restoration by synaptic dysfunction by BMS-708163 and Nilotinib in our AD model.

## Methods

### Cell culture and neural differentiation

Human ESC, KhES-1 and PS1-overexpressing hESCs derived from KhES-1 were cultured and differentiated as described previously^26^,^48^. Briefly, undifferentiated hESCs were cultured on mitomycin C-treated mouse embryonic fibroblasts in primate ES medium (ReproCELL, Japan), supplemented with 5 ng/ml fibroblast growth factor 2 (Wako Chemical Co., Japan). The use of the hESC lines conformed to the Guidelines for Derivation and Utilization of Human Embryonic Stem Cells of the Ministry of Education, Culture, Sports, Science, and Technology (MEXT) of Japan. We used a single clone each of PS1-WT and PS1-G378E hESC lines because there was no variation among any PS1-WT clones and among any PS1-G378E clones in the protein and gene expression levels^26^ and the drug response to a γ-secretase inhibitor, DAPT (Supplementary Fig. S3).

The neural differentiation method has been previously described^26^. Briefly, 100 nM LDN-193189 (Cellagen Technology) and 1 μM SB431542 (Sigma-Aldrich) were used for the neural induction of hESCs. Neurons differentiated from hESCs were cultured in neuron maintenance medium: N2B27 medium supplemented with 10 ng/ml brain-derived neurotrophic factor (BDNF; PeproTech), 10 ng/ ml glial cell-derived neurotrophic factor (GDNF; PeproTech), 10 ng/ml neurotrophin-3 (NT-3; PeproTech) and 100 ng/mL beta-nerve growth factor (NGF; R&D Systems). There was no difference in the neural differentiation efficiency between PS1-WT and PS1-G378E hESC lines (approximately 80% NeuN-positive cells)^26^.

### Chemical screening

The chemical screening procedure is described in Fig. 2a. Human ESC-derived neurons were treated with 1 μM cytosine arabinoside (AraC) on day 7 to eliminate dividing cells. By treatment with AraC, the numbers of Ki67-positive cells reduced to less than approximately 11%. After 3 days of treatment, the cells were replated and cultured in neuron maintenance medium with DMSO (1/20,000) as a negative control, 800 nM DAPT (Calbiochem) as a positive control, or each chemical (5 μM) per well for 10 days. We used two chemical libraries, the SCREEN-WELL FDA-approved drug library V2 Japan version (ENZO life sciences) and the Bioactive Compound Library (Selleck chemicals). The culture media were harvested for the measurement of Aβ levels. Cell viability was measured using a cell proliferation reagent (WST-1; Roche). The ratios of Aβ40 and the cell viability in each chemical treatment were relative to those in DMSO treatment. The relative Aβ40 ratio was adjusted based on the cell viability. The hit chemicals in the first screening were determined by the following criteria: the average Aβ40 ratios were less than the average ratio – 2 SD (mean – 2 SD). The same culture conditions were used for the second chemical screening (5 μM). In the second screening, the compounds causing a greater than 50% reduction compared to DMSO treatment were considered as hit compounds. The dose-dependent effects of Nilotinib, Pimecrolimus, Rosuvastatin Calcium, Sulconazole Nitrate salt and Toremifene Base (all from ENZO life sciences) (0, 0.001, 0.01, 0.1, 1, and 10 μM) were investigated using neurons derived from KhES-1.

### Chemical treatment

For the chemical treatment experiments, neurons derived from hESCs were cultured without replating for 30 days. For the isolation of synaptosomes and the patch-clamp experiments, hESC-derived neurons were cultured in neuron maintenance medium with each chemical from day 10 to 30. BMS-708163 (Selleck chemicals) (10 nM), DAPT (Calbiochem) (800 nM), Nilotinib (Cayman Chemical), Pimecrolimus (Cayman Chemical), Rosuvastatin Calcium (Tokyo Chemical Industry Co., Japan), Sulconazole Nitrate salt (SANTA CRUZ) and Toremifene Base (LKT Laboratories) (5 μM) were used.

### Amyloid-β 40 measurement

In the chemical screening, AlphaScreen reagent and a Human Amyloidβ 1-40 (High Specificity) immunoassay kit (PerkinElmer) were used with an Enspire multimode plate reader (PerkinElmer). Human Amyloid β (1-40) (FL) Assay Kits (IBL, Japan) were used for the other experiments.

### Isolation of synaptosomes

Synaptosomes containing functional synaptic proteins were isolated from neurons that had been cultured for 30 days using a Syn-PER Synaptic Protein Extraction Reagent (Thermo Scientific) according to the manual. For the Western blotting analysis, the synaptosome fraction was dissolved in RIPA buffer supplemented with 5 mM EDTA and 1× Halt Protease and Phosphatase Inhibitor Cocktail (both from Thermo Scientific). The protein concentrations were measured using a Pierce BCA Protein Assay Kit (Thermo Scientific). The enrichment of synaptosomes was confirmed by the relative enhancement of the levels of a pre-synaptic protein (SNAP25) compared to that in whole cell extract (Supplementary Fig. S4).

### Western blotting

The Western blot analyses were carried out as previously described^26^. PVDF membranes were incubated with the following primary antibodies: anti-beta actin (1:5000), anti-DBN (drebrin) (1:300), anti-MUNC13-4 (1:800), anti-RAB3A (1:200), anti-SV2A (1:300), anti-SV2C (1:300), anti-SYNG1 (synaptogyrin 1) (1:600), anti-SYT1 (synaptotagmin 1) (1:250) (all from Abcam), anti-NSF (1:750), anti-SNAP25 (1:400) (Cell Signaling Technology), anti-RAB3C (1:1000) (Synaptic Systems), anti-RPH3A (rabphilin 3A) (1:1000) (Santa Cruz), anti-SV2B (1:150), anti-SYP (synaptophysin) (1:200) (Sigma-Aldrich) or anti-SYN1 (synapsin 1) (1:200) (Millipore). Anti-rabbit IgG-HRP (Santa Cruz) or anti-mouse IgG-HRP (Cell Signaling Technology) were used as secondary antibodies. The target proteins were detected with the Novex ECL HRP Chemiluminescent Reagent Kit (Invitrogen) and an ImageQuant LAS 4000 system (FUJI FILM).

### Quantitative reverse transcription polymerase chain reaction (qRT-PCR)

Total RNA was extracted from hESC-derived neurons that had been cultured for 28 days using an RNeasy mini kit (QIAGEN) and was subjected to cDNA synthesis using the SuperScript III Reverse Transcriptase (Invitrogen). A qRT-PCR was carried out using SYBR Premix Ex Taq (Takara Bio Inc., Japan) and StepOnePlus (Applied Biosystems). The relative amount of each gene was normalized against β-actin. The primers used in the qRT-PCR were purchased from Takara Bio Inc.

### The patch-clamp method

An electrophysiological analysis was performed as described previously^26^. Human ESC-derived neurons that had been cultured with BMS-708163, DAPT, Nilotinib or Pimecrolimus were randomly selected and voltage-clamped at −70 mV. The current events that occurred in a 60-second period were recorded as sEPSCs.

### Statistical analysis

Statistical significance was determined by the Mann-Whitney U test for differences between two groups, and by Steel’s test for comparisons of three or more groups. p values of <0.05 were considered to indicate statistical significance.

## Additional Information

**How to cite this article**: Nishioka, H. *et al*. BMS-708163 and Nilotinib restore synaptic dysfunction in human embryonic stem cell-derived Alzheimer’s disease models. *Sci. Rep.* **6**, 33427; doi: 10.1038/srep33427 (2016).

## Supplementary Material

Supplementary Information
