# Supplementary material for: BMS-708163 and Nilotinib restore synaptic dysfunction in human embryonic stem cell-derived Alzheimer’s disease models
Source: Sci Rep. 2016 Sep 19;6:33427. doi: 10.1038/srep33427 (PMC5027582; doi:10.1038/srep33427)
Supplement: Supplementary Information [file srep33427-s1.pdf]

Supplementary information

BMS-708163 and Nilotinib restore synaptic dysfunction in human embryonic stem cell-derived Alzheimer's disease models

Hisae Nishioka[1,2], Norie Tooi[1], Takehisa Isobe[1], Norio Nakatsuji [1,3], Kazuhiro Aiba [1, 4*]

[1]Institute for Integrated Cell-Material Sciences (WPI-iCeMS), Kyoto University, Kyoto 606-8501, Japan

[2]Graduate School of Medicine, Kyoto University, Kyoto 606-8501, Japan

[3] Institute for Frontier Medical Sciences, Kyoto University, Kyoto 606-8507, Japan

[4]Current address: Stem Cell & Device laboratory, Inc., Kyoto 600-8491, Japan

[*]Corresponding Author: Kazuhiro Aiba

Institute for Integrated Cell-Material Sciences (WPI-iCeMS), Kyoto University

Yoshida-Ushinomiyacho, Sakyo-ku, Kyoto 606-8501, Japan

Tel: +81-75-753-9762

Fax: +81-75-753-9785

E-mail: kaiba@icems.kyoto-u.ac.jp

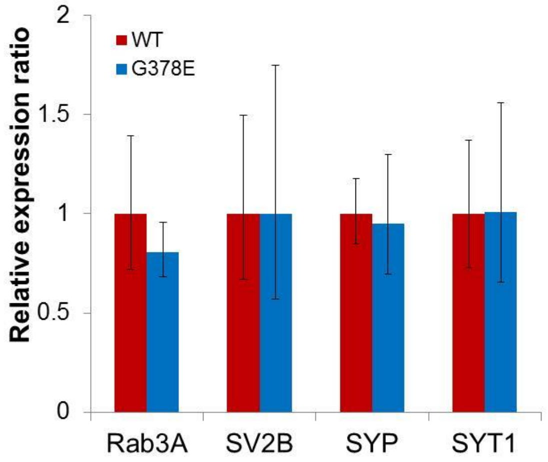

Supplementary Figure S1. **PS1-G378E neurons did not show significant differences in RAB3A and SV2B gene expression levels**

Gene expression levels of RAB3A, SV2B, Synaptophysin (SYP) and Synaptotagmin 1 (SYT1) were indicated in PS1-G378E neurons (G378E) relative to PS1-WT neurons (WT). β-actin was used as an internal control. Each gene expression level in PS1-WT neurons was defined as 1.0. Mann–Whitney U test was used to check for differences in expression levels. Four independent experiments, each time in triplicates were performed (n = 4). Mean ± SD.

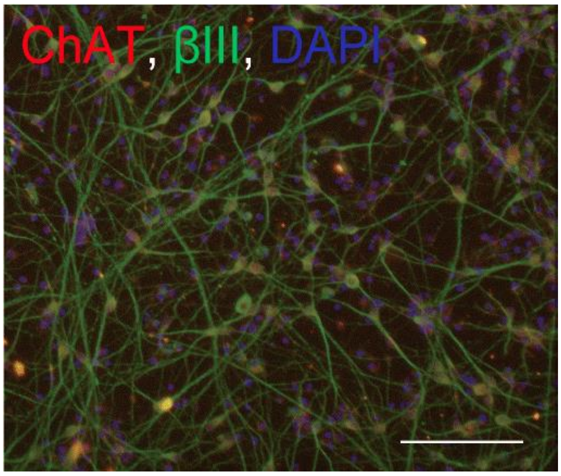

Supplementary Figure S2. **Cholinergic neurons derived from PS1-overexpressing hESCs.**

Immunocytochemistry using antibodies against a cholinergic neuron marker, choline acetyltransferase (ChAT, red) and a neuron marker, βIII-tubulin (βIII, green) were carried out. Cells were counterstained with 4′,6-diamidino-2-phenylindole (DAPI, blue) to visualize nuclei. Scale bar, 100 μm. A few cells (0.9 ± 0.7%) were detected as choline acetyltransferase-positive neurons in hESC-derived neurons used in this study.

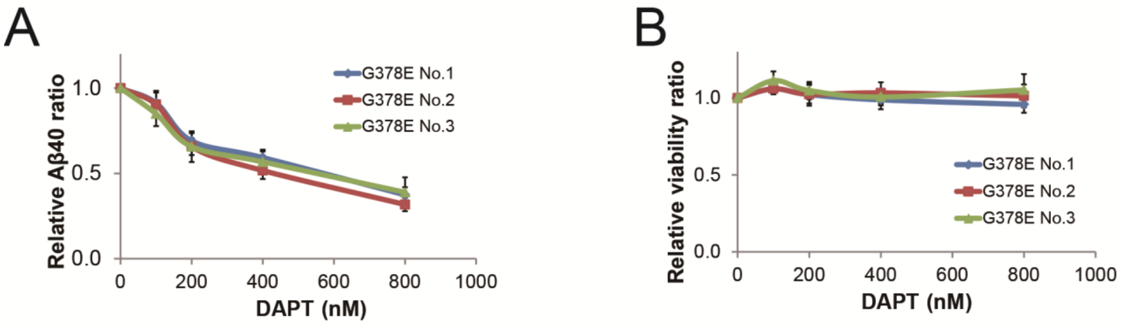

Supplementary Figure S3. **Drug responses and cell viability of PS1-G378E neurons derived from three subclones expressing mutant-PS1**

The effects of Aβ inhibition (A) and cell survival (B) in the presence of various concentrations of DAPT using three subclones of PS1-G378E neurons. The amount of Aβ40 and cell viability in DMSO-treated PS1-G378E neurons was defined as 1.0. Three independent experiments, each time in triplicates were performed (n = 3). Mean ± SD.

Using 3 subclones (No.1 – 3) of the hESCs overexpressing PS1-G378E (PS1-G378E hESCs), we investigated whether there is any variation among neurons derived from the PS1-G378E hESC subclones. In our previous report, it was confirmed that the PS1 protein expression levels were not significantly different between different subclones[1]. DAPT-response experiments showed that there were also no differences in DAPT responses (Aβ40 reduction and cell viability) among the subclones (Supplementary Fig. S2). These data indicate that there are no significant variations among PS1-G378E subclones. In addition, all of clones had an identical genetic background because the site-specific gene integration method was applied for establishing clones overexpressing mutant PS1. Hence we used each single clone of PS1-G378E neurons for the experiments in this study.

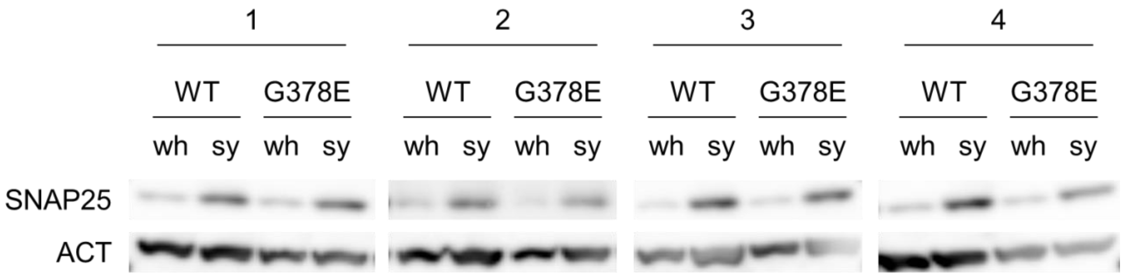

Supplementary Figure S4. **Preparation of synaptosomes from the PS1-WT and PS1-G378E neurons**

Neural differentiation, following synaptosome preparation were independently carried out four times (n = 4). Immunoblot analyses of a pre-synaptic protein, SNAP25 were performed using the whole cells and synaptosomes of PS1-WT and PS1-G378E neurons. These data indicated that isolation of synaptosomes was successfully carried out in all preparation. β-actin (ACT) was used as an internal control. WT, PS1-wild type neurons; G378E, PS1-G378E neurons; wh, whole cells; sy, synaptosomes.

Supplementary Table S1. **Result of chemical screening**

	Chemicals	Relative Aβ40 ratios*		Relative viability ratios (2nd screening)**
		1st screening	2nd screening	
γ-secretase inhibitor	Semagacestat (LY-450139)[2]	0.088	-	-
	Avagacestat (BMS-708163)[3]	0.050	-	-
	DAPT(GSI-IX)[4]	0.14	-	-
	LY-411575[5]	0.23	-	-
	MK-0752[6]	0.10	-	-
	YO-01027 (Dibenzazepine)[7]	0.11	-	-
Bcr-Abl inhibitor	Nilotinib[8]	0.21	0.23	0.85
Calcineurin inhibitor	Pimecrolimus	0.20	0.27	0.87
HMG-CoA reductase inhibitor	Fluvastatin Sodium[9]	0.28	0.37	0.96
	Rosuvastatin Calcium	0.18	0.19	0.91
Imidazole derivative	Sulconazole Nitrate salt	0.23	0.25	1.04
Selective estrogen receptor modulator	Toremifene Base	0.27	0.23	0.92

*, The Aβ40 level of DMSO treatment was considered to be 1.0.

**, The cell viability ratio of DMSO treatment was considered to be 1.0.

Supplementary Table S2. **The numerical data of figure 2d and 2e**

	Relative Aβ40 ratios (Mean±SD)*				
	K1 + chemicals				
chemicals (μM)	Nilo	Pime	Rosu	Sulc	Tore
0.001	0.96±0.21	0.80±0.20	0.95±0.16	0.90±0.12	0.79±0.17
0.01	0.89±0.13	0.69±0.15	0.85±0.10	0.78±0.17	0.86±0.10
0.1	0.93±0.06	0.75±0.07	0.72±0.17	0.87±0.15	0.77±0.17
1	0.71±0.12	0.74±0.13	0.72±0.36	0.63±0.21	0.63±0.20
10	0.24±0.10	0.52±0.17	0.39±0.07	0.30±0.14	0.14±0.06

	Relative viability ratios (Mean±SD)**				
	K1 + chemicals				
chemicals (μM)	Nilo	Pime	Rosu	Sulc	Tore
0.001	1.16±0.10	1.05±0.09	0.97±0.18	1.04±0.19	1.19±0.25
0.01	1.19±0.23	1.07±0.15	0.93±0.11	1.19±0.22	1.02±0.09
0.1	1.09±0.09	0.99±0.11	0.94±0.12	0.98±0.19	1.05±0.19
1	1.05±0.18	1.02±0.10	0.84±0.18	1.11±0.12	0.97±0.22
10	0.62±0.14	0.86±0.22	0.72±0.14	0.72±0.12	0.67±0.22

*, The Aβ40 level of DMSO treatment was considered to be 1.0.

**, The cell viability ratio of DMSO treatment was considered to be 1.0.

K1, KhES-1-derived neurons; Nilo, Nilotinib; Pime, Pimecrolimus; Rosu, Rosuvastatin Calcium; Sulc, Sulconazole Nitrate; Tore, Toremifene Base.

References

1 Honda, M. *et al.* The modeling of Alzheimer's disease by the overexpression of mutant Presenilin 1 in human embryonic stem cells. *Biochem Biophys Res Commun* **469**, 587-592 (2016).

2 Lanz, T. A. *et al.* Concentration-Dependent Modulation of Amyloid-β in Vivo and in Vitro Using the γ-Secretase Inhibitor, LY-450139. *J Pharmacol Exp Ther* **319**, 924-933 (2006).

3 Gillman, K. W. *et al.* Discovery and Evaluation of BMS-708163, a Potent, Selective and Orally Bioavailable γ-Secretase Inhibitor. *ACS Med Chem Lett* **1**, 120-124 (2010).

4 Lanz, T. A. *et al.* The γ-Secretase Inhibitor N-[N-(3,5-Difluorophenacetyl)-l-alanyl]-S-phenylglycine t-butyl Ester Reduces Aβ Levels in Vivo in Plasma and Cerebrospinal Fluid in Young (Plaque-Free) and Aged (Plaque-Bearing) Tg2576 Mice. *J Pharmacol Exp Ther* **305**, 864-871 (2003).

5 Lanz, T. A. *et al.* Studies of Aβ Pharmacodynamics in the Brain, Cerebrospinal Fluid, and Plasma in Young (Plaque-Free) Tg2576 Mice Using the γ-Secretase Inhibitor N2-[(2S)-2-(3,5-Difluorophenyl)-2-hydroxyethanoyl]-N1-[(7S)-5-methyl-6-oxo-6,7-dihydro-5H-dibenzo[b,d]azepin-7-yl]-L-alaninamide (LY-411575). *J Pharmacol Exp Ther* **309**, 49-55 (2004).

6 Cook, J. J. *et al.* Acute γ-Secretase Inhibition of Nonhuman Primate CNS Shifts Amyloid Precursor Protein (APP) Metabolism from Amyloid-β Production to Alternative APP Fragments without Amyloid-β Rebound. *J Neurosci* **30**, 6743-6750 (2010).

7 Milano, J. *et al.* Modulation of Notch Processing by γ-Secretase Inhibitors Causes Intestinal Goblet Cell Metaplasia and Induction of Genes Known to Specify Gut Secretory Lineage Differentiation. *Toxicol Sci* **82**, 341-358 (2004).

8 Lonskaya, I. *et al.* Nilotinib-induced autophagic changes increase endogenous parkin level and ubiquitination, leading to amyloid clearance. *J Mol Med* **92**, 373-386 (2014).

9 Kurinami, H. *et al.* Prevention of amyloid beta-induced memory impairment by fluvastatin, associated with the decrease in amyloid beta accumulation and oxidative stress in amyloid beta injection mouse model. *Int J Mol Med* **21**, 531-537 (2008).
